# VEGF-A related SNPs: a cardiovascular context

**DOI:** 10.3389/fcvm.2023.1190513

**Published:** 2023-05-23

**Authors:** J. C. Meza-Alvarado, R. A. Page, B. Mallard, C. Bromhead, B. R. Palmer

**Affiliations:** School of Health Sciences, Massey University, Wellington, New Zealand

**Keywords:** vascular endothelial growth factor, single nucleotide polymorphism, cardiovascular disease, VEGF-A eQTLs, genetic association

## Abstract

Cardiovascular diseases (CVDs) are the leading cause of death worldwide. Currently, cardiovascular disease risk algorithms play a role in primary prevention. However, this is complicated by a lack of powerfully predictive biomarkers that could be observed in individuals before the onset of overt symptoms. A key potential biomarker for heart disease is the vascular endothelial growth factor (VEGF-A), a molecule that plays a pivotal role in blood vessel formation. This molecule has a complex biological role in the cardiovascular system due to the processes it influences, and its production is impacted by various CVD risk factors. Research in different populations has shown single nucleotide polymorphisms (SNPs) may affect circulating VEGF-A plasma levels, with some variants associated with the development of CVDs, as well as CVD risk factors. This minireview aims to give an overview of the VEGF family, and of the SNPs reported to influence VEGF-A levels, cardiovascular disease, and other risk factors used in CVD risk assessments.

## Introduction

Cardiovascular diseases (CVDs) are defined by the World Health Organization as a group of disorders that affect the heart and blood vessels in terms of structure or blood supply (1). Notable examples of CVDs that are a leading cause of death globally include coronary heart disease (CHD), acute coronary syndrome (ACS) and congenital heart disease (2). CHD involves inadequate coronary blood supply, which may arise from a blockage in the coronary arteries usually following progressive narrowing of the lumen of atherosclerotic blood vessels (3). Given the multifactorial nature of CVDs, there are reviews available that explore in greater detail specific diseases such as coronary artery disease (CAD) (4), CHD (5), the underlying mechanism of atherosclerosis (6, 7) and the relationship of these diseases with specific variables (8, 9).

Overall, risk factors for CVDs can be grouped as modifiable or non-modifiable. The modifiable risk factors involve lifestyle circumstances that can be behavioral (diet, physical activity, exercise, smoking, alcohol) or metabolic (circulating lipid levels and glucose levels) in nature (8). Whereas, age, genetics and ethnicity of individuals are the non-modifiable risk factors. This distinction informs the diagnosis of CVDs by determining which critical variables should be included in CVD risk assessments. Critical factors employed have included age, hypercholesterolemia, high density lipoprotein (HDL) cholesterol levels, gender, smoking, diabetes, and systolic blood pressure (10).

Genetic determinants are important non-modifiable risk factors for CVDs that have been studied intensively since the early 21st century (11–13). The influence of genetic factors on CVD development was initially explored through family history studies focused on single gene disorders during the 1980 s (4, 14). Most CVDs are now considered to be polygenic disorders impacted by susceptibility and disease-linked genes, with major impacts from lifestyle and environmental factors (14). Susceptibility genes are associated with an increase or reduction in the risk of developing a disease. Comparatively, disease-linked genes are those whose expression is linked to a pathological phenotype (4). Both susceptibility and disease-linked genes can influence the regulation of other genes and/or factors that are directly involved in the pathobiology of different CVDs. The genetic basis for CVDs such as CAD and CHD has been reviewed in greater detail elsewhere (11, 15–17).

**Figure 1:**
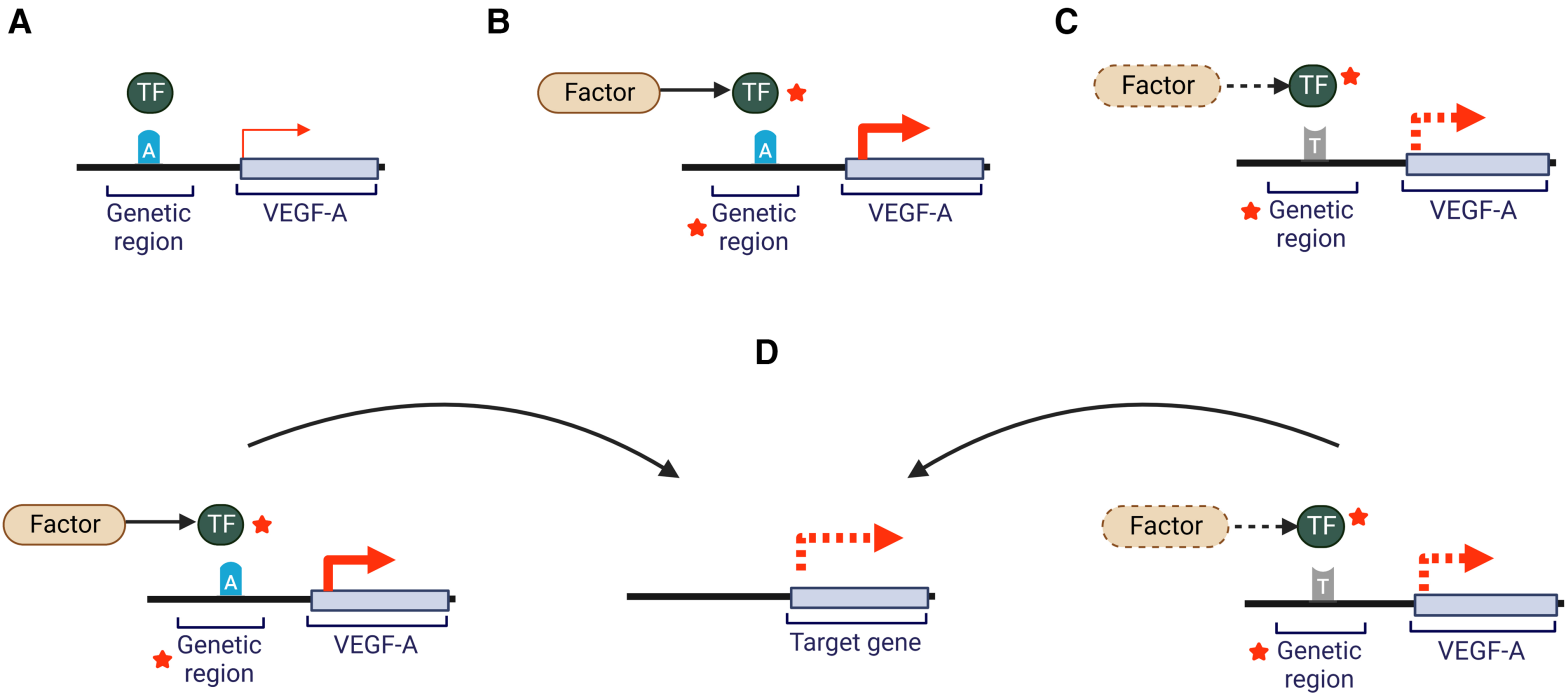
Potential effects of a SNP on VEGF-A gene expression. Gene regions where a SNP may be located include gene promoters, enhancers, introns, transcription factor binding motifs or non-coding RNAs. Dotted lines indicate hypothetical changes that could decrease or increase interactions or activity. Red arrows represent VEGF-A expression. Red stars indicate potential for an increase or decrease in activity through a variety of molecular mechanisms (**A**) an individual carrying the common allele “A” will show normally regulated levels of VEGF-A. (**B**) In the presence of a risk factor (e.g., elevated LDL-cholesterol plasma levels) and the common allele “A” there may be altered activity of genomic regions or transcription factors leading to altered VEGF-A expression and potentially impact circulating VEGF-A levels and/or activity. (**C**) Presence of a non-normal range risk factor (e.g., elevated plasma glucose levels) and the alternate allele “T”, may impact activity of genomic regions or transcription factors leading to altered VEGF-A expression and potentially influence VEGF-A levels and/or activity (**D**) The changes illustrated in scenario B and C may impact expression of genes located at a distance, which may impact other pathways (e.g., lipid metabolism). Adapted from (29). Created with BioRender.com.

Considering this genetic complexity, numerous studies have focused on identifying associations between genetic variants and common cardiovascular disease traits (15, 18–21). This has been supported with the establishment of genome wide association studies (GWAS), which employ technologies that detect many gene variants simultaneously (22). The predominant variants identified through GWAS are single nucleotide polymorphisms (SNPs) (15, 22–24). SNPs can be located within a protein-coding region, where they may display a functional effect, but they can also be in non-coding and regulatory areas of the genome (e.g., introns, enhancer, etc.). Moreover, SNPs can play a regulatory role by impacting gene expression and protein concentration if they are located within genetic elements such as transcription factor binding sites, splicing regions, enhancer, promoter, or silencer regions (23, 25). These are often called expression quantitative trait loci (eQTLs) and explain a proportion of the genetic variance of a particular phenotype (26). SNPs can also influence coding regions located within the same loci (cis-acting) or interact with coding regions of other chromosomes or distant loci on the same chromosome (trans-acting) (27, 28). Specifically, SNP variants can influence CVD risk through traditional risk factors, such as plasma lipid levels and blood pressure (11, 27, 28). Overall, SNP variants can have several potential effects on any given gene as summarized in Figure 1. One example covered in this review is *VEGFA,* which impacts the cardiovascular system through angiogenesis and increased endothelial cell activity.

Coupling our understanding of CVD pathogenesis with associations of regulatory SNPs with coronary biomarkers, there is potential for the combined use of CVD-relevant genetic risk scores (cvdGRS) in risk prevention (30). This involves using multiple SNPs identified from GWAS studies in different populations and these SNP variants can be associated with clinical outcomes or risk factors (30, 31). Overall, the goal of cvdGRS is to aid in patient risk stratification and treatment (22, 28, 31–33). The functional effects of the SNP variants may provide evidence to underpin a clinical framework for prevention, treatment, and in severe cases, genetic counselling in primary care (22, 31, 34, 35).

## VEGF overview

A molecule of interest in the development and progression of CVDs is the vascular endothelial growth factor (VEGF-A), a member of the platelet-derived growth factor (PDGF)/VEGF family (36, 37). This growth factor is involved in blood vessel formation, with reported impacts on the development of CVDs, as well as potential recovery (38, 39). VEGF-A was originally referred to as a vascular permeability factor, with activity observed in tumor cells from rodents (40). In 1989 several research groups identified that this factor selectively promoted the migration of vascular endothelium and induced angiogenesis *in vivo* (41–43). Based on these findings, factors with this activity were renamed and classified as members of the VEGF family (43).

The VEGF family are glycoproteins expressed under the regulation of soluble mediators such as growth factors or cytokines (39, 44, 45). They are involved in the regulation of blood vessel formation through endothelial cell differentiation or from existing blood vessels (44, 46). Additionally, the VEGF family is involved in lymphangiogenesis, endothelial cell survival and vascular permeability regulation, amongst other functions (44, 47). However, alterations in their functionality have also been associated with the development of atherosclerosis, CHD, tumor formation, neovascularization, and other pathologies including cancer, diabetic retinopathy, preeclampsia, and endometriosis (23, 39, 47, 48).

There are five VEGF family members that directly influence the human cardiovascular system. The archetype member is VEGF-A, a potent stimulator of vasculogenesis and angiogenesis (44, 48, 49). VEGF-A production is influenced by oxygen tension, hormones (e.g., estrogen) and proinflammatory cytokines (47, 49, 50). VEGF-B induces the development of the cardiovascular system, embryonic angiogenesis and the formation of embryonic myocardium as well as participating in blood vessel survival (51). VEGF-C and VEGF-D are primarily involved in lymphangiogenesis, while the placental growth factor (PIGF) participates in both angiogenesis and wound healing (39, 47, 49).

These VEGF proteins act through one or more of three tyrosine kinase VEGF receptors (VEGFRs) found on the surface of endothelial and non-endothelial cells (44). VEGFR1 (Flt-1) and VEGFR2 (KDR) participate in angiogenesis. VEGFR2 is the primary inducer of VEGF-mediated blood vessel growth, while VEGFR3 is involved in lymphangiogenesis (47, 52, 53). Additionally, VEGFR1 has the co-receptor neuropilin-1 (NRP1), which selectively potentiates VEGFR2-mediated vascular permeability, and endothelial cell motility in vascular development (49, 54). Once activated, the signaling pathways of these receptors have the downstream effect of influencing vascular tone, blood vessel formation, endothelial cell proliferation and migration (47). VEGFR signaling is reported to also be activated in a non-VEGF-dependent manner through receptor phosphorylation due to shear stress, or recognition of alternative ligands such as lactate and low-density lipoproteins (LDLs) (36, 53, 55).

Specifically, the VEGF-A canonical pathway occurs when it binds to either VEGFR1 or VEGFR2. This promotes receptor homodimerization or heterodimerization that leads to the phosphorylation of the receptor's intracellular domains (53, 55). VEGFR1 has a soluble splice variant (sFlt-1) that acts as a decoy receptor, decreasing VEGF-A plasma concentration and limiting its binding to KDR (36, 44, 47). Also, VEGF-A activity can be potentiated when PIGF displaces it from VEGFR1 to VEGFR2 (39). These and other mechanisms surrounding the regulation of VEGF receptors have been reviewed in greater detail elsewhere (37, 55).

## *VEGFA* gene and related SNPs in a cardiovascular context

**Figure 2:**
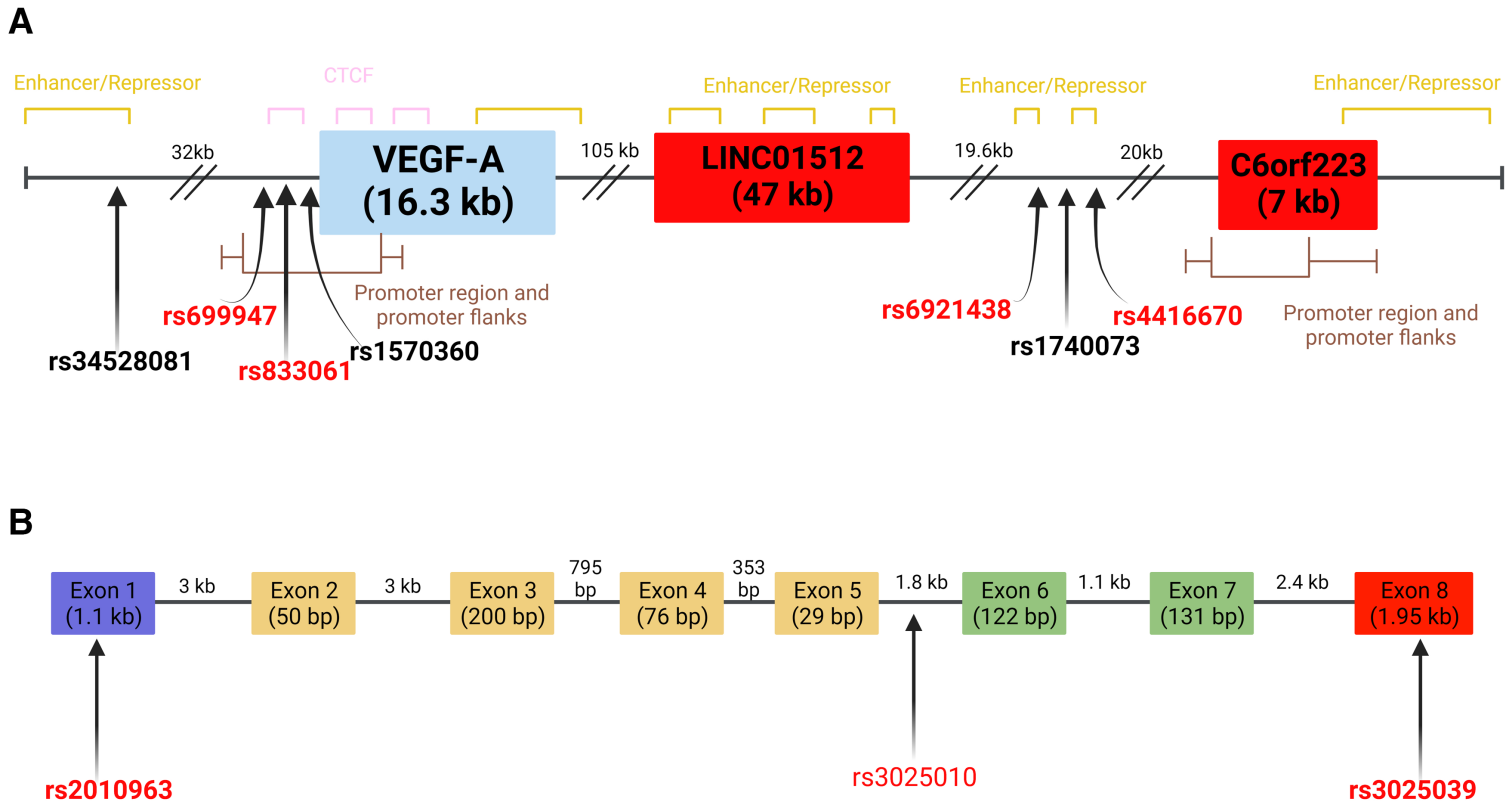
*VEGFA* gene and select SNPs on chromosome 6 (**A**) location of VEGF-A related SNPs at chromosome 6 loci. SNPs in bold have been reported to influence VEGF-A levels. SNPs in red have reported associations to CVD risk factors or biomarkers. Red boxes indicate regions that represent lncRNA. Full gene length is indicated individually. Distances between SNPs and genes are indicated above dashes. (**B**) Schematic representation of SNPs located within exons and introns of *VEGFA*. Individual exon and intron lengths are shown (65). The purple exon contains the signal sequence of the gene. The red exon confers anti-angiogenic capabilities when present. Green exons contain heparin-binding domains with extracellular matrix components. Yellow exons are involved in VEGF receptor binding. Location of SNPs and genomic regions determined using data from the UCSC genome browser and Ensembl databases (GRCh38-/hg38). Created with BioRender.com.

The *VEGFA* gene has a 16.3 kb coding region located at 6p21.1 on the long arm of chromosome 6, including eight exons and seven introns (56, 57). The first five exons are constitutively present among VEGF-A isoforms, since they encode the signal sequence for protein processing and residues that bind to VEGF receptors (54, 58). Meanwhile, exons 6 and 7 contain the heparin binding domains that allow some isoforms to bind to cell surfaces and impact their activity or bioavailability depending on which are present (59, 60). Lastly, exon 8 undergoes post-translational readthrough due to a non-canonical stop codon, leading to the production of sub-exons 8a and 8b, with the latter being reported to be present in a unique isoform with anti-angiogenic activity observed in bone disorders and brain diseases (54, 61–63). So far, 16 distinct VEGF-A isoforms have been identified (47, 54). The different isoforms depend on the presence or absence of exons 6 and 7, which affect the affinity for heparin or heparan sulfate proteoglycans. For example, the most prevalent VEGFA isoform is VEGFA_165_, which lacks exon 6, but has moderate heparin affinity allowing the isoform to remain bound to cell surfaces (64). Comparatively the isoform subtype VEGFA_121_ lacks exon 6 and 7 so it is found only in free form (64). Despite their size difference most of the VEGF-A isoforms act as endothelial cell mitogens, upregulate the endothelial expression of adhesion molecules and present pro-angiogenic activity (36, 51, 64). Pathologies caused by increased angiogenesis include inflammatory diseases, cancers, retinopathy and atherosclerosis, while reduced angiogenesis has been observed in bone disorders and brain diseases (61). The overall *VEGFA* gene structure including SNPs with reported influence on VEGF-A expression levels (discussed below and in Supplementary Tables S1, S2) is presented in Figure 2.

Altered plasma and tissue levels of VEGF-A have been observed in various conditions including ischemic heart disease (IHD), CAD, strokes, heart failure, and myocardial infarction (38, 66–68). Due to its impact on angiogenic processes, the effect of high VEGF-A circulating levels on CVD onset varies. High VEGF-A levels are associated with various CVD risk factors including smoking, hypercholesterolemia, diabetes, hypertension, and hyperglycemia (36). Additionally, increased VEGF-A activity has been associated with inflammation, increased blood pressure and an increase in the formation of atherosclerotic lesions, leading to CHD (20, 69–71). The impact of angiogenic molecules on atherosclerosis has been reviewed elsewhere (39).

Expression of *VEGFA* can be upregulated by the hypoxia inducible factor, p53 allele polymorphisms, thyroid stimulating hormone, estrogen levels and oxygen tension (45, 47). This matches studies that show VEGF-A production is influenced by elements associated with atherosclerosis including LDL concentration, hypoxia, and interleukin activity (38, 49, 72). The increased production of VEGF-A can negatively impact human health by influencing the development of atherosclerotic plaques, by affecting vascular dilation, adhesion protein expression, monocyte migration, endothelium permeability and increased trans-endothelial lipid migration (38, 39). High levels of VEGF-A in plasma have been associated with increased plaque growth and subsequent lesion vulnerability that can cause intraplaque hemorrhage (73). There is evidence that proinflammatory cytokines (e.g., IL-1, IL-6, and IL-18) present during CVD onset can enhance VEGF-A production, thus exacerbating atherosclerotic lesion development (74–76).

VEGF-A is considered a highly polymorphic gene because of the 148 untranslated region (UTR), 209 exon, 779 intron, and 124 near-gene variants that have been identified (77). At least 30 SNPs within the untranslated, exon, intron and promoter regions may have the potential to influence variation in VEGF-A expression (78, 79). This genetic influence over VEGF-A circulating levels has been explored in various studies. Debette et al. (80) investigated the heritability of VEGF-A levels in healthy individuals without a cancer diagnosis. This study identified four common variants (rs6921438, rs4416670, rs6993770 and rs10738760) distributed across three chromosomes that were independently associated with circulating VEGF-A levels and explained up to 48% of the heritability of serum VEGF-A levels (80). A meta-analysis of GWAS data evaluated the association of variants with circulating VEGF-A levels (81). Choi et al. (81) found a total of ten SNPs contributed up to 52% of the variability in circulating VEGF-A levels with some SNPs associated with increased or decreased VEGF-A levels compared to median. Additional information on the study details of SNPs identified by these groups and other studies are presented in Supplementary Tables S1, S2. The Supplementary Material also includes SNPs that have been studied in relation to VEGF-A levels in healthy individuals, CVDs, or comorbidities related to the risk of CVD (e.g., diabetes, metabolic syndrome, hypertension).

Some of the SNPs that have been studied are located within exonic regions of *VEGFA* (82)*.* One noteworthy eQTL is rs2010963 from exon 1 of *VEGFA* (Figure 2B). The CC genotype has been associated with increased VEGF-A levels in type 2 diabetes mellitus (T2DM) (83, 84). Furthermore, the rs2010963 CC genotype has been linked to risk factors including heart rate (83), blood glucose levels (77), blood pressure, cholesterol and HDL levels (83, 85). There is also evidence for this variant influencing VEGF-A levels in non-CVDs such as glioma (86) and diabetic retinopathy (48, 87).The variant rs3025039, located within exon 8 of *VEGFA,* has similar effects (Figure 2B). Dong et al. (88) observed that patients diagnosed with gestational diabetes mellitus carrying the TT genotype had higher levels of VEGFA compared to healthy pregnant women (88). Meanwhile Ruggiero et al. (89) reported that the TT genotype was associated with lower median levels of VEGFA in healthy population samples from villages in Southern Italy. Some studies showed the CT genotype of rs3025039 is associated with reduced VEGFA levels as well as reducing risk of presenting with CHD and T2DM (77, 89). The associations reported for the rs3025039 variant demonstrate its link to the cardiovascular system, but the variety of findings suggest additional studies are needed. An additional variant that has shown association to the cardiovascular system is rs3025010 located in intron 5 (Figure 2B). In a Chinese cohort diagnosed with hypertension the C allele of this variant was observed to be associated with lower systolic and diastolic blood pressure measurements (20). Furthermore, in a Chinese case-control study, it was observed that the CC genotype of rs3025010 reduced risk of brain arteriovenous malformation (91). This evidence shows a clear link to CVD risk which could be further explored in additional ethnic groups to validate or identify other biomarker associations.

Other variants of interest can be found at the same loci, but outside the intron and exon regions of *VEGFA* (25, 80). rs69214328, is located within an enhancer region found between two long non-coding RNA genes (Figure 2A). The GWAS findings of Choi et al. (81) and Debette et al. (80) identified that the A allele of rs69214328 is associated with lower serum levels of the VEGF-A protein. Additionally, the same allele has also been reported to influence the variability of HDL and LDL in individuals of European ancestry (92). The A allele of rs6921438 appears to have eQTL activities since increased serum levels of IL-6, TNF-α and VEGF-A were observed in interaction with SNPs rs6993770 (Chr8), rs4416670 (Chr6) and rs10738760 (Chr9), respectively (93). Two additional variants (rs1740073 and rs34528081) located on chromosome 6 (Figure 2A) were identified by Choi et al. (81) to be associated with serum levels of VEGF-A (Supplementary Table S1). Furthermore, the T allele of rs34528081 was observed to be associated with increased VEGF-A serum levels in an additional GWAS study (Supplementary Table S1). Meanwhile, the T allele of rs1740073 has been reported to associate with increased VEGF-A serum in a GWAS study while analysis of IHD using 1,000 Genomes European data reported that the same allele could contribute to VEGF variance (66).

Another variant that has been studied is rs699947, which is located in the promoter region of *VEGFA* (Figure 2A). Various groups report that the AA genotype of rs699947 is associated with increased risk in cardiovascular pathologies including CAD, CHD, stroke and congenital heart diseases (Supplementary Table S1). The A allele of rs699947 has been associated with total cholesterol, LDL and apolipoprotein B (77, 83, 94). These associations have been observed across different ethnic groups, which further suggests rs699947 is a potential genetic risk marker for CVDs (89, 95, 96). For its part, rs833061 is another variant that is located within the promoter region of *VEGFA* (Figure 2A) whose CT genotype has been observed to reduce VEGF-A levels in a T2DM cohort (77). Other reports have also shown this variant is associated with hypertension and a meta-analysis of 3 cohorts implies this variant can influence congenital heart disease risk in individuals of Asian ancestry (Supplementary Table S1). A variant located further from the promoter region that presents a similar array of findings related to lipid metabolism and inflammatory molecules is rs4416670 (Figure 2A). Both its alleles have been linked to CVD risk factors and biomarkers (Supplementary Table S1). Specifically, the T allele was reported by Debette et al. (80) to increase VEGF-A serum levels while a study by Azimi-Nezhad et al. (93) reported the same allele could decrease IL-6 levels by interacting with rs6921438 (Chr6) and rs10738760 (Chr9). However, Azimi-Nezhad et al. (93) also report that the C allele of rs4416670 can increase TNF-α and IL-6 levels by interacting with the A allele of rs6921438 thus implying a link between both VEGF-A related SNPs and inflammatory molecules. Additionally, the C allele has also been observed in other studies to be associated with apolipoprotein E levels, hypertension and metabolic syndrome (92, 97). These findings demonstrate links between VEGF-A related SNPs and lipid metabolism, inflammatory biomarkers and CVD risk factors.

Some gene variants have findings of associations with molecules used in CVD risk assessment. For example, the rs1570360 variant located in the promoter region of VEGF-A (Figure 2A), was observed to contribute to an increased risk of congenital heart disease (98). Some reports showed that the GA genotype of this variant is associated with a reduced left ventricular ejection fraction and extracranial internal carotid artery (ECICA) stenosis which are both risk factors for systemic hypertension and ischemic stroke, respectively (Supplementary Table S1). However, in a Chinese study the GG genotype was observed to increase susceptibility for coronary heart disease in patients with high smoking habits and diagnosed with hypertension. As such, this variant shows consistent links to CV risk factors which, given its location, could be attributed to a potential influence on VEGF as observed in variants located within the promoter region (rs699947 and rs833061).

Similar studies have been reported for other SNPs located across the genome, often denoted as trans-acting SNPs (Supplementary Table S2). Broadly, these eQTL SNPs have been associated with increased risk of CVDs (e.g., CAD, CHD, IHD) (66, 89, 99, 100) or metabolic syndrome (81). One example rs1870377, located on chromosome 4 in exon 11 of the *VEGFR2* (*KDR*) gene (Supplementary Figure S1) can influence cardiovascular outcomes. Li et al. (72) reported that the AA genotype reduces risk of unfavorable CVD outcomes, particularly those related with disability, in an Asian ancestry cohort. Marks et al. (99) also reported that the AA genotype associated with reduced risk of heart failure readmission and the A allele associated with high levels of VEGF system components, specifically sFlt-1 and KDR (101), and increased the risk of ischemic stroke in a Korean cohort (100). The TA and TT genotypes were both associated with increased CHD prevalence in Han Chinese populations (89, 99). Location of additional SNPs influencing VEGF-A expression levels within the *VEGFR2* (*KDR*) gene is presented in Supplementary Figure S1. Additional associations observed for trans-acting SNPs are presented in Supplementary Table S2.

rs6993770 is located on chromosome 8 in intron 4 of the *ZPFM2* gene, which codes for a protein involved in heart morphogenesis and coronary vessel development. Broadly, studies on this variant have shown relationships with VEGF-A, CVD and CVD risk factors (Supplementary Table S2). In the GWAS findings of Choi et al. (81) and the Mendelian Randomization study done by Au Yeung (66), the A allele correlated with increased VEGF-A serum levels. The GWAS findings of Debette et al. (80) showed the T allele was associated with increased VEGF-A serum levels. Other studies involving individuals of European and Iranian ancestry observed the T allele was also associated with risk biomarkers of CVD, particularly fasting blood glucose, triglyceride levels, systolic blood pressure and HDL levels (92, 102). The TA genotype has been reported to increase the risk of metabolic syndrome (102), and impacts the expression of adhesion molecules (ICAM-1, E-selectin) as well as IL-6 levels (93). Meanwhile, the TT genotype appears to contribute to metabolic syndrome risk in individuals with low iron intake (97). This spectrum of reports demonstrates the range of associations that alleles and genotypes of trans-acting SNPs, such as rs1870377 or rs6993770, may have within the cardiovascular system. Additional trans-acting SNPs (e.g., rs2071559, rs114694170, rs6993770, rs10738760, rs10761741, rs4782371 have been reported to be capable of influencing VEGF-A circulating levels (81) or soluble VEGFR levels (rs1870377) (101). Specific study details and overall findings are presented in Supplementary Table S2. Notably, two SNPs (rs2305948 and rs7667298) have associations with potential CVD risk, but their direct impact on VEGF system components was observed in cancer related studies (103, 104). Interestingly, trans-acting SNPs most likely involve interactions with molecules or homeostatic mechanisms that have known roles in CVD onset, including inflammatory interleukins (70, 93), triglycerides, adhesion molecules, blood cell count and blood pressure (66, 102, 105, 106). There are cases of specific variants that correlate with increased risk of presenting major adverse coronary events (rs2305948, rs7667298) (106), CHD (rs2305948, rs1870377, rs2071559, rs7667298) (89, 99), ischemic stroke (rs1870377) (100) and metabolic syndrome (rs6993770) (102). As such, some SNPs appear to be potential contributors to phenotypes (IHD, CAD, CHD) while others may increase or reduce disease risk depending on the presence or absence of risk factors (72, 89, 101).

## Conclusion

Overall, the impact of VEGF-A related SNPs in various forms of heart disease has been explored in many different types of studies. The collective evidence reveals a critical subset of cis-acting SNPs mapping to the region of *VEGFA* (Figure 2 and Supplementary Table S1)*,* several trans-acting SNPs mapping in the region of the *VEGFR2* gene (Supplementary Figure S1) and elsewhere on the human genome (Supplementary Table S2), with repeatable associations with circulating levels of VEGF-A. A small group of SNPs reproducibly associate with established biomarkers and risk factors for heart disease (rs2010963, rs3025039, rs1570360, rs699947, rs6921438) or with increased susceptibility to common heart disease pathologies (rs2010963, rs3025039, rs1570360, rs699947, rs2305948, rs1870377). This minireview highlights that these SNPs can be potential markers for CVDs and may influence significant biological pathways that impact the cardiovascular system (e.g., lipid metabolism). The wide range of pathologies that VEGF-A and its related SNPs impact emphasizes the complexity of VEGF-A interactions within the cardiovascular system. Both cis- and trans-acting SNP eQTLs can affect expression levels, but there remain many unknowns around the specific mechanisms involved. There is a clear link between SNPs and VEGF-A levels as well as established cardiovascular disease biomarkers (HDL, LDL, BNP, NTproBNP). Together these have the potential to act synergistically on the development of CVDs.

The complexity of SNP influences on CVD and CVD risk factors reinforces the importance of studying them in relation to VEGF-A. Particularly considering how altered levels of VEGF-A contribute to disease onset or exacerbate an individual's health depending on the risk factors they present with. Exploring the link between CVDs, SNPs, and VEGF-A may contribute to improved cardiovascular disease risk assessment, prevention, treatment, and. prognosis.
