# Supplementary material for: VEGF-A related SNPs: a cardiovascular context
Source: Front Cardiovasc Med. 2023 May 23;10:1190513. doi: 10.3389/fcvm.2023.1190513 (PMC10242119; doi:10.3389/fcvm.2023.1190513)
Supplement: Supplementary file 1 [file Image1.pdf]

Supplementary Material

VEGF-A related SNPs: a cardiovascular context

Meza-Alvarado, J.C.,[1] Page R.A,[1] Mallard B.[1], Bromhead C.[1] Palmer, B.R.[1]
*** Correspondence:** B.R. Palmer b.palmer@massey.ac.nz

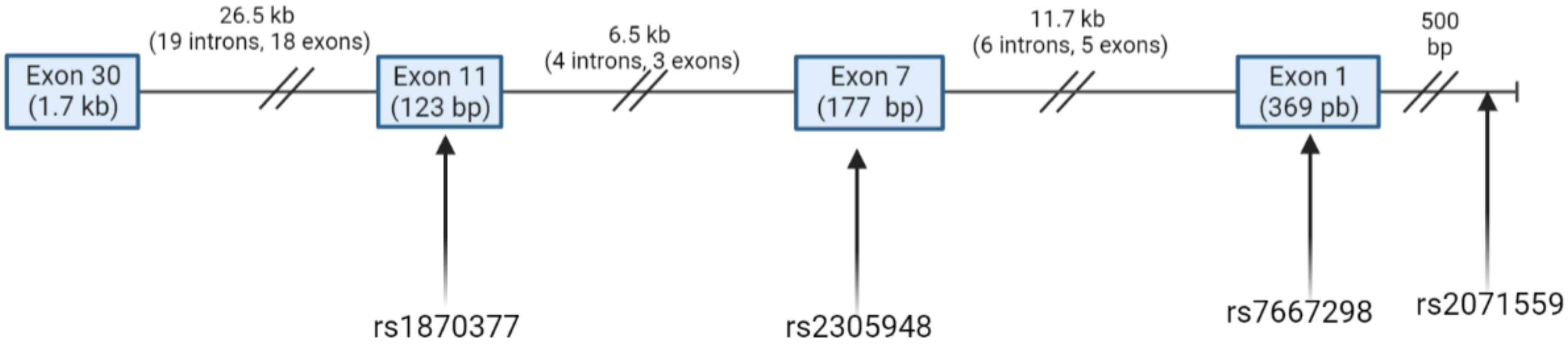

Supplemental Figure 1. Schematic representation of key SNPs in key exons of VEGFR2 (Chromosome 4, reverse strand). Full exon length is indicated individually. Distances between exon and SNPs are indicated above dashes. Distance measured using NCBI GenBank Accession NC_000004
