# Supplementary material for: VEGF-A related SNPs: a cardiovascular context
Source: Front Cardiovasc Med. 2023 May 23;10:1190513. doi: 10.3389/fcvm.2023.1190513 (PMC10242119; doi:10.3389/fcvm.2023.1190513)
Supplement: Supplementary file 2 [file Table1.pdf]

# *Supplementary Material*

# VEGF-A related SNPs: a cardiovascular context

Meza-Alvarado, J.C.,[1] Page R.A,[1] Mallard B.[1], Bromhead C.[1] Palmer, B.R.[1]
*** Correspondence:** B.R. Palmer b.palmer@massey.ac.nz

## Supplementary Table 1. VEGF-A eQTLS on Chromosome 6

| SNP | Location* | Study type | Population (n) | Disease studied | Associated Biomarkers[$] | Associated risk factors[$] | Variant Effect or Association | Genotype or Allele | Source |
|---|---|---|---|---|---|---|---|---|---|
| **rs34528081 (- →T)** | Chr6: 43736681 | GWAS Meta-analysis | 10 cohorts of European ancestry (n = 16,112) | Effect on VEGF levels | VEGF-A | N/A | Increased serum VEGF-A levels (9 of 10 cohorts) | T allele | [1] |
| | | GWAS community-based study | 3 healthy cohorts from USA (n = 3,527), France (n = 859), and Sweden (n = 868) | Effect on VEGF levels | VEGF-A | N/A | Increased serum VEGF-A levels (2 study groups) | T allele | [2] |
| **rs2010963 (G>C)** | Chr6: 43770613 (+405 G/C) | Cross-sectional case-control study | Polish participants ($n_{cases}$ = 265, $n_{controls}$ = 158) | Excess body mass | Estimated glomerular filtration rate | BMI | Negative correlation of VEGF-A serum levels with biomarkers | GG genotype | [3] |
| | | | | | Heart rate, diastolic blood pressure and HDL serum levels | N/A | Positive correlation of VEGF-A serum levels with biomarkers | CC genotype | |
| | | Cohort study | Unrelated Han Chinese patients (n = 242) | Coronary Artery Disease | N/A | N/A | Potential influence on CAD | CC genotype | [4] |

| | | | | | | | | | |
|---|---|---|---|---|---|---|---|---|---|
| | | Case-control study | Iranian participants ($n_{cases}$ = 347, $n_{controls}$ = 173) | Coronary Artery Disease | N/A | N/A | Increased susceptibility to CAD | CC genotype | [5] |
| | | Case-control study | Brazilian participants ($n_{cases}$ = 169, $n_{controls}$ = 179) | Systemic hypertension | N/A | Left ventricular mass index | N/A | GC and CC genotypes | [6] |
| | | Meta-analysis of case-control studies | 10 cohorts: 6 Asian ($n_{cases}$ = 1826, $n_{controls}$ = 1946) and 4 Caucasian ($n_{cases}$ = 477, $n_{controls}$ = 916) | Coronary Artery Disease and Myocardial Infarction | N/A | N/A | Increased risk of CAD | CC genotype | [7] |
| | | Cross-sectional study | Slovenian participants ($n_{cases}$ = 595, $n_{controls}$ = 200) | Type 2 Diabetes Mellitus | N/A | N/A | Higher VEGF-A serum levels in T2DM patients. | CC genotype | [8] |
| | | Retrospective case-control study | Unrelated adults of Tunisian Arab origin ($n_{cases}$ = 815, $n_{controls}$ = 805) | Type 2 Diabetes Mellitus | Serum glucose, VEGF-A | Age | Reduced risk of T2DM Increased VEGF levels | GC genotype | [9] |
| | | Cohort study | Unrelated Mexican mestizo participants (n = 415) | Type 2 Diabetes Mellitus | Diastolic blood pressure, total cholesterol, and HDL | N/A | N/A | Both alleles | [10] |

| | | | | | | | | | |
|---|---|---|---|---|---|---|---|---|---|
| | | Case-control study | Unrelated Chinese participants ($n_{cases}$ = 533, $n_{controls}$ = 533) | Coronary heart disease | N/A | Increased risk of CHD | N/A | GG genotype | [11] |
| | | Case-control study | Unrelated Han Chinese participants ($n_{cases}$ = 319, $n_{controls}$ = 333) | Hemorrhagic stroke | N/A | Increased risk of brain arterio-venous malformation | N/A | CC genotype | [12] |
| **rs3025039 (C>T)** | Chr 6:43784799 (+936 C/T) | Meta-analysis of case-control studies | 8 cohorts: 4 Asian ($n_{cases}$ = 1626, $n_{controls}$ = 1681) and 4 Caucasian ($n_{cases}$ = 510, $n_{controls}$ = 796) | Coronary Artery Disease and Myocardial Infarction | N/A | Asian ancestry | Increased risk of CAD | TT genotype | [7] |
| | | Cohort study | New Zealand patients including 3 ethnic groups: European, Māori and Pasifika (n = 1927) | Acute coronary syndromes | Collateral vessel perfusion, BNP and NT-proBNP | N/A | Increased VEGF-A levels | TT genotype | [13] |
| | | Retro-spective case-control study | Unrelated adults of Tunisian Arab origin ($n_{cases}$ = 815, $n_{controls}$ = 805) | Type 2 Diabetes Mellitus | Diabetes duration, HbA1c, serum triglycerides, VEGF-A | N/A | Reduced T2DM risk and reduced VEGF levels | CT genotype | [9] |
| | | Population-based | Italian (n = 1957) | Influence on VEGF levels | VEGF-A | N/A | Lower median serum VEGF levels | TT genotype | [14] |

| | | | | | | | | |
|---|---|---|---|---|---|---|---|---|
| | | cohort study | | | | | | |
| | | Hospital-based case-control study | Korean ($n_{cases}$ = 650, $n_{controls}$ = 308) | Ischemic stroke | Apolipoprotein B | Stroke ad Extracranial internal carotid artery stenosis | Increased risk of stroke | T allele | [15] |
| | | Meta-analysis | 9 Asian cohorts ($n_{cases}$ = 1565, $n_{controls}$ = 2551) | Congenital heart diseases | N/A | N/A | Increased risk of tetralogy of Fallot | N/A | [16] |
| | | Case-control study | Unrelated Han Chinese participants ($n_{cases}$ = 810, $n_{controls}$ = 805) | Coronary Heart Disease | N/A | Smoking, alcohol intake, diabetes | Reduced risk of presenting CHD | CT genotype | [17] |
| | | Case-control study | Chinese ($n_{cases}$ = 239, $n_{controls}$ = 275) | Gestational diabetes mellitus | VEGF-A | Increased risk of GDM | Higher VEGF-A expression levels | CT and TT genotypes | [18] |
| **rs1570360 (G>A)** | Chr6:43770093 (-1154 G/A) | Case-control study | Brazilian participants ($n_{cases}$ = 169, $n_{controls}$ = 179) | Systemic hypertension | N/A | Reduced ejection fraction | N/A | GA and AA genotypes | [6] |
| | | Retrospective case-control study | Unrelated adults of Tunisian Arab origin ($n_{cases}$ = 815, $n_{controls}$ = 805) | Type 2 Diabetes Mellitus | Serum glucose | N/A | Increased risk of T2DM | A allele | [9] |
| | | Hospital-based case-control study | Korean ($n_{cases}$ = 650, $n_{controls}$ = 308) | Ischemic stroke | Total homocysteine levels | Extracranial internal carotid artery (ECICA) stenosis | N/A | GA genotype | [15] |

| | | | | | | | | | |
|---|---|---|---|---|---|---|---|---|---|
| | | Meta-analysis | 5 cohorts: 3 Asian ($n_{cases}$ = 455, $n_{controls}$ = 670) and 2 Caucasian ($n_{cases}$ = 248, $n_{controls}$ = 368) | Congenital heart diseases | N/A | N/A | Increased risk of congenital heart disease | N/A | [16] |
| | | Case-control study | Unrelated Han Chinese participants ($n_{cases}$ = 810, $n_{controls}$ = 805) | Coronary Heart Disease | N/A | Smoking, Hypertension | Increased susceptibility to CHD | GG genotype | [17] |
| **rs699947 (C>A)** | Chr6:43768652 (−2578 C/A) | Cross-sectional case-control study | Polish participants ($n_{cases}$ = 265, $n_{controls}$ = 158) | Excess body mass | HDL, VEGF-A | BMI | Positive correlation of VEGF-A serum levels with biomarkers | CC genotype | [3] |
| | | | | Normal weight | Total cholesterol, LDL, VEGF-A | BMI | Negative correlation of VEGF-A serum levels with biomarkers | AA genotype | |
| | | Cohort study | Unrelated Han Chinese patients (n = 242) | Coronary Artery Disease | N/A | N/A | N/A | AA genotype | [4] |
| | | Meta-analysis of case-control studies | 8 cohorts: 5 Asian ($n_{cases}$ = 2062, $n_{controls}$ = 2113) and 3 Caucasian ($n_{cases}$ = 409, $n_{controls}$ = 698) | Coronary Artery Disease and Myocardial Infarction | N/A | Asian ancestry | Increased risk of CAD | AC genotype | [7] |
| | | Cohort study | New Zealand patients including 3 ethnic | Acute Coronary Syndrome | N/A | Lower physical activity | Predictor of mortality in male non-diabetic participants | AA genotype | [13] |

| | | | | | | | | | |
|---|---|---|---|---|---|---|---|---|---|
| | | | groups: European, Māori and Pasifika (n = 2067) | | | | | | |
| | | Retrospective case-control study | Unrelated adults of Tunisian Arab origin ($n_{cases}$ = 815, $n_{controls}$ = 805) | Type 2 Diabetes Mellitus | Serum glucose, LDL | N/A | Increased risk of T2DM | A allele | [9] |
| | | Hospital-based case-control study | Korean ($n_{cases}$ = 650, $n_{controls}$ = 308) | Ischemic stroke | Total homocysteine levels, Apolipoprotein B | Extracranial internal carotid artery stenosis | Increased risk of stroke | A allele | [15] |
| | | Case-control study | Unrelated Han Chinese participants ($n_{cases}$ = 810, $n_{controls}$ = 805) | Coronary Heart Disease | N/A | Hypertension, Diabetes, alcohol intake | Increased risk of CHD | AA genotype | [17] |
| **rs1740073 (T>C)** | Chr6:43979661 (Intergenic between *LINC0512* and *C6orf223*) | GWAS Meta-analysis | 10 cohorts of European ancestry (n = 16,112) | Effect on VEGF levels | VEGF-A | N/A | Increased serum VEGF-A levels (8 of 10 cohorts) | T allele | [1] |
| | | Mendelian random-ization study | 1000 Genomes data on adults of European ancestry ($n_{cases}$ = 60,801 $n_{controls}$ = 123,504) | Ischemic Heart Disease | VEGF-A | N/A | Potential contributor to VEGF phenotypic variance | T allele | [19] |

| | | | | | | | | | |
|---|---|---|---|---|---|---|---|---|---|
| **rs6921438 (G>A)** | Chr6:43957870 (Intergenic between *LINC0512* and *C6orf223*) | GWAS Meta-analysis | 10 cohorts of European ancestry (n = 16,112) | Effect on VEGF levels | VEGF-A | N/A | Lower serum VEGF-A levels (10 cohorts) | A allele | [1] |
| | | Mendelian random-ization study | 1000 Genomes data on adults of European ancestry ($n_{cases}$ = 60,801 $n_{controls}$ = 123,504) | Ischemic Heart Disease | HDL, LDL and VEGF-A | N/A | Increased serum VEGF levels | G allele | [19] |
| | | Population -based phenome wide association study | Finnish participants (n = 6,890) | Inflammatory biomarker driver trait search | VEGF-A, IL-10. IL-12p70 | N/A | Estimated effect on VEGF protein production | N/A | [20] |
| | | GWAS community-based study | 3 healthy cohorts from USA (n = 3,527), France (n = 859), and Sweden (n = 868) | Effect on VEGF levels | VEGF-A | N/A | Lower serum VEGF-A levels (2 study groups) | A allele | [2] |
| | | Population study | 2 groups of unrelated healthy European ancestry ($n_1$ = 1,006 $n_2$ = 1,145) | Influence on lipid metabolism | HDL, LDL | N/A | Contributes to 1% of HDL variability and 0.2% of LDL variability | A allele | [21] |
| | | Case-control study | Iranian participants ($n_{cases}$ = 248, $n_{controls}$ = 100) | Association between dietary intake and metabolic Syndrome | Zinc and manganese intake | Metabolic Syndrome | Association of SNP with low iron intake and high manganese intake. | AA genotype | [22] |
| | | Population study | Healthy French | Association between | IL-6, TNF-α, VEGF-A | N/A | Epistatic interaction with two other SNPs is | A allele | [23] |

| | | | | | | | | | |
|---|---|---|---|---|---|---|---|---|---|
| | | | individuals (n = 403) | VEGF, adhesion, and inflammation molecules. | | | associated with increased IL-6 levels. Another interaction with two individual SNPs is associated with increased VEGF-A and TNF-α levels. | | |
| | | | | | IL-6 | N/A | Epistatic interaction with two other SNPs is associated with decreased IL-6 levels. | G allele | [23] |
| **rs4416670 (T>C)** | Chr6:43982716 (Intergenic between *LINC0512* and *C6orf223*) | GWAS community-based study | 3 healthy cohorts from USA (n = 3,527), France (n = 859), and Sweden (n = 868) | Effect on VEGF levels | VEGF-A | N/A | Increased serum VEGF-A levels (2 study groups) | T allele | [2] |
| | | Population study | 2 groups of unrelated healthy European ancestry ($n_1$ = 1,006 $n_2$ = 1,145) | Influence on lipid metabolism | Apolipoprotein E | Hypertension | Potential functional effect between variant, hypertension and ApoE levels. | C allele | [21] |
| | | Case-control study | Iranian participants ($n_{cases}$ = 248, $n_{controls}$ = 100) | Association between dietary intake and metabolic Syndrome | Iron, copper, zinc, manganese, and iodine intake | Metabolic Syndrome | Increased risk of Metabolic syndrome | CT and CC genotypes | [22] |
| | | Population study | Healthy French individuals (n = 403) | Association between VEGF, adhesion, and inflammation molecules. | IL-6, TNFα | N/A | Epistatic interaction with one SNP is associated with increased TNF-α levels. Another interaction with two other SNPs is associated with increased IL-6 levels. | C allele | [23] |

| | | | | | | | | | |
|---|---|---|---|---|---|---|---|---|---|
| | | | | | IL-6 | | Epistatic interaction with two SNPs is associated with decreased IL-6 levels | T allele | [23] |
| **rs3025010 (C>T)** | Chr6:43779840 (Intron 5) | Cross-sectional population study | Chinese ($n_{cases}$ = 258, $n_{controls}$ = 258) | Hypertension | N/A | Lower systolic and diastolic blood pressure | N/A | C allele | [24] |
| | | Case-control study | Unrelated Han Chinese participants ($n_{cases}$ = 319, $n_{controls}$ = 333) | Haemo-rrhagic stroke | N/A | Reduced risk of brain arterio-venous malformation | N/A | CC genotype | [12] |
| **rs833061 (C>T)** | Chr6:43769749- (460 T/C) | Retro-spective case-control study | Unrelated adults of Tunisian Arab origin ($n_{cases}$ = 815, $n_{controls}$ = 805) | Type 2 Diabetes Mellitus | VEGF-A | N/A | Reduced VEGF-A serum levels | CT genotype | [9] |
| | | Cross-sectional population study | Chinese ($n_{cases}$ = 258, $n_{controls}$ = 258) | Hypertension | N/A | Hypertension | N/A | N/A | [24] |
| | | Meta-analysis | 3 cohorts: 2 Asian ($n_{cases}$ = 233, $n_{controls}$ = 318) and 1 Caucasian ($n_{cases}$ = 102, $n_{controls}$ = 112) | Congenital heart diseases | N/A | Asian ancestry | Influence on congenital heart disease risk | N/A | [16] |

*Location reported with point location (Ensembl human database GRCh38.p13) specifying the variant's genomic context and where applicable indicating nomenclature in reference to the VEGF-A gene

$ Biomarkers or risk factors associated to the allele or genotype reported by each study

N/A: Not reported by the article

ApoE: Apolipoprotein E, BMI: Body Mass Index, BNP: B-type natriuretic peptide, CAD: Coronary Artery Disease, CHD: Coronary Heart Disease, eQTLs: expression quantitative trait loci, GWAS: Genome Wide Association Study, HbA1c: Haemoglobin A1C, HDL: High Density Lipoprotein, IL: Interleukin, LDL: Low Density Lipoprotein, N/A: not applicable, NT-proBNP: N-terminal pro B-type natriuretic peptide, SNP: Single Nucleotide Polymorphism, T2DM: Type 2 Diabetes Mellitus, TNF: Tumour Necrosis Factor, USA: United States of America, VEGF: Vascular Endothelial Growth Factor

**References**


1. Choi, S.H., et al., *Six Novel Loci Associated with Circulating VEGF Levels Identified by a Meta-analysis of Genome-Wide Association Studies.* PLOS Genetics, 2016. **12**(2): p. e1005874.
2. Debette, S., et al., *Identification of cis- and trans-acting genetic variants explaining up to half the variation in circulating vascular endothelial growth factor levels.* Circulation Research, 2011. **109**(5): p. 554-63.
3. Skrypnik, D., et al., *Association of rs699947 (-2578 C/A) and rs2010963 (-634 G/C) Single Nucleotide Polymorphisms of the VEGF Gene, VEGF-A and Leptin Serum Level, and Cardiovascular Risk in Patients with Excess Body Mass: A Case-Control Study.* Journal of Clinical Medicine, 2020. **9**(2).
4. Cui, Q.T., et al., *Further evidence for the contribution of the vascular endothelial growth factor gene in coronary artery disease susceptibility.* Gene, 2013. **521**(2): p. 217-221.
5. Nia, S.K., et al., *The impact of vascular endothelial growth factor+405 C/G polymorphism on long-term outcome and severity of coronary artery disease.* Journal of Clinical Laboratory Analysis, 2017. **31**(4).
6. Lacchini, R., et al., *Effect of Genetic Polymorphisms of Vascular Endothelial Growth Factor on Left Ventricular Hypertrophy in Patients With Systemic Hypertension.* The American Journal of Cardiology, 2014. **113**(3): p. 491-496.
7. Ma, W.-Q., et al., *Association of genetic polymorphisms in vascular endothelial growth factor with susceptibility to coronary artery disease: a meta–analysis.* BMC Medical Genetics, 2018. **19**(1).
8. Merlo, S., et al., *Vascular Endothelial Growth Factor Gene Polymorphism (rs2010963) and Its Receptor, Kinase Insert Domain-Containing Receptor Gene Polymorphism (rs2071559), and Markers of Carotid Atherosclerosis in Patients with Type 2 Diabetes Mellitus.* Journal of Diabetes Research, 2016.
9. Sellami, N., et al., *Association of VEGFA variants with altered VEGF secretion and type 2 diabetes: A case-control study.* Cytokine, 2018. **106**: p. 29-34.
10. Totomoch-Serra, A., et al., *Association of common polymorphisms in the and genes with type 2 diabetes-related traits in Mexicans.* Archives of Medical Science, 2018. **14**(6): p. 1361-1373.
11. Li, L., et al., *Association of Genetic Polymorphisms on Vascular Endothelial Growth Factor and its Receptor Genes with Susceptibility to Coronary Heart Disease.* Medical Science Monitor, 2016. **22**: p. 31-40.
12. Chen, H., et al., *Polymorphisms of the vascular endothelial growth factor A gene and susceptibility to sporadic brain arteriovenous malformation in a Chinese population.* Journal of Clinical Neuroscience, 2011. **18**(4): p. 549-553.
13. Palmer, B.R., et al., *Vascular endothelial growth factor-A promoter polymorphisms, circulating VEGF-A and survival in acute coronary syndromes.* PLOS One, 2021. **16**(7).
14. Ruggiero, D., et al., *Genetics of VEGF serum variation in human isolated populations of cilento: importance of VEGF polymorphisms.* PLOS One, 2011. **6**(2): p. e16982.
15. Yadav, B.K., et al., *Genetic Polymorphisms rs699947, rs1570360, and rs3025039 on the VEGF gene are correlated with extracranial internal carotid artery stenosis and ischemic stroke.* Annals of Clinical and Laboratory Science, 2017. **47**(2): p. 144-155.

16. Wang, W., A. Xu, and H. Xu, *The roles of vascular endothelial growth factor gene polymorphisms in congenital heart diseases: a meta-analysis.* Growth Factors, 2018. **36**(5-6): p. 232-238.
17. Liu, D., et al., *Association of Genetic Polymorphisms on VEGFA and VEGFR2 With Risk of Coronary Heart Disease.* Medicine, 2016. **95**(19): p. e3413.
18. Dong, P.-P., *Association of vascular endothelial growth factor expression and polymorphisms with the risk of gestational diabetes mellitus.* Journal of Clinical Laboratory Analysis, 2019. **33**(2): p. e22686-e22686.
19. Au Yeung, S.L., H. Lam, and C.M. Schooling, *Vascular Endothelial Growth Factor and Ischemic Heart Disease Risk: A Mendelian Randomization Study.* Journal of the American Heart Association, 2017. **6**(8).
20. Ruotsalainen, S.E., et al., *An expanded analysis framework for multivariate GWAS connects inflammatory biomarkers to functional variants and disease.* European Journal of Human Genetics, 2021. **29**(2): p. 309-324.
21. Stathopoulou, M.G., et al., *A common variant highly associated with plasma VEGFA levels also contributes to the variation of both LDL-C and HDL-C.* Journal of Lipid Research, 2013. **54**(2): p. 535-41.
22. Hoseini, Z., et al., *VEGF gene polymorphism interactions with dietary trace elements intake in determining the risk of metabolic syndrome.* Journal of Cellular Biochemistry, 2019. **120**(2): p. 1398-1406.
23. Azimi-Nezhad, M., et al., *Associations of vascular endothelial growth factor (VEGF) with adhesion and inflammation molecules in a healthy population.* Cytokine, 2013. **61**(2): p. 602-7.
24. Zhao, Z., et al., *Association between Single Nucleotide Polymorphisms in Cardiovascular Developmental Critical Genes and Hypertension: A Propensity Score Matching Analysis.* International Journal of Hypertension, 2020: p. 1-8.
