# Supplementary material for: VEGF-A related SNPs: a cardiovascular context
Source: Front Cardiovasc Med. 2023 May 23;10:1190513. doi: 10.3389/fcvm.2023.1190513 (PMC10242119; doi:10.3389/fcvm.2023.1190513)
Supplement: Supplementary file 3 [file Table2.pdf]

# *Supplementary Material*

## VEGF-A related SNPs: a cardiovascular context

Meza-Alvarado, J.C.,[1] Page R.A,[1] Mallard B.[1], Bromhead C.[1] Palmer, B.R.[1]
*** Correspondence:** B.R. Palmer b.palmer@massey.ac.nz

### Supplementary Table 2. Reports on trans-acting SNPs

| SNP (change)* | Location (proximal gene/s) * | Gene function$ | Study type | Population | Disease Studied | Notable findings | Source |
|---|---|---|---|---|---|---|---|
| **rs2305948 (C>T)** | Chr4: 55113391 (*VEGFR2*) | VEGF receptor | Case-control study | Unrelated Chinese participants ($n_{cases}$ = 533, $n_{controls}$ = 533) | Coronary heart disease | Increased CHD risk (TT genotype) | [1] |
| | | | Case-control study | Unrelated Han Chinese participants ($n_{cases}$ = 810, $n_{controls}$ = 805) | Coronary Heart Disease | Reduced risk to CHD when smoking, alcohol intake and diabetes were considered (TT genotype) | [2] |
| | | | Meta analysis of case-control studies | 10 cohorts: 8 Asian and 2 Caucasian ($n_{cases}$ = 5,474 $n_{controls}$ = 8,584) | Atherosclerotic cardiovascular diseases (CAD, MI, IS) | This SNP is associated with coronary artery disease | [3] |
| | | | Cohort study | Han Chinese patients undergoing coronary intervention (n = 275) | Coronary Heart Disease | Higher presence of main adverse cardiovascular events including angina pectoris and recurrent myocardial infarction (TT genotype) | [4] |
| **rs1870377 (A>T)** | Chr4: 55106807 (*VEGFR2*) | VEGF receptor | Case-control study | Unrelated Chinese participants ($n_{cases}$ = 533, $n_{controls}$ = 533) | Coronary heart disease | Increased CHD risk (TT genotype) | [1] |
| | | | Cohort Study | New Zealand patients including 3 ethnic groups: European, Māori and Pasifika (n = 2067) | Acute coronary syndrome | Increased levels of sFlt-1 and sKDR (A allele). There is an association with reduced risk of heart failure remission (AA genotype) | [5] |

| | | | | | | | |
|---|---|---|---|---|---|---|---|
| | | | Meta analysis of case-control studies | 10 cohorts: 8 Asian and 2 Caucasian ($n_{cases}$ = 5,474 $n_{controls}$ = 8,584) | Atherosclerotic cardiovascular diseases (CAD, MI, IS) | This SNP is associated with coronary artery disease and ischaemic stroke | [3] |
| | | | Case-control study | Unrelated Han Chinese participants ($n_{cases}$ = 810, $n_{controls}$ = 805) | Coronary Heart Disease | Increased CHD risk in non-hypertensive, nondiabetic, and non-smoking cases (TA genotype) | [2] |
| | | | Cohort study | Han Chinese patients (n = 1,016) | Large Artery Atherosclerotic Stroke | Decreased risk of unfavourable outcomes (AA genotype) | [6] |
| | | | Case-control study | Korean participants ($n_{cases}$ = 501, $n_{controls}$ = 478) | Ischemic Stroke | Confers risk of presenting ischemic stroke (A allele) | [7] |
| **rs7667298 (A>G)** | Chr4: 55125564 (*VEGFR2*) | VEGF receptor | Case-control study | Unrelated Han Chinese participants ($n_{cases}$ = 810, $n_{controls}$ = 805) | Coronary Heart Disease | Increased CHD risk in non-hypertensive, nondiabetic, and non-smoking patients (G allele) | [2] |
| | | | Cohort study | Han Chinese patients undergoing coronary intervention (n = 275) | Coronary Heart Disease | Lower occurrence of target lesion revascularization (GG genotype) | [4] |
| **rs2071559 (T>C)** | Chr4: 55126199 (*VEGFR2*) | VEGF receptor | Cross-sectional study | Slovenian participants ($n_{cases}$ = 595, $n_{controls}$ = 200) | T2DM | Higher VEGF-A serum levels in T2DM patients (CC genotype). The SNP also shows association with carotid intima-media thickness in T2DM patients | [8] |
| | | | Case-control study | Unrelated Chinese participants ($n_{cases}$ = 533, $n_{controls}$ = 533) | Coronary heart disease | Increased CHD risk (CC genotype) | [1] |
| | | | Meta analysis of case-control studies | 10 cohorts: 8 Asian and 2 Caucasian ($n_{cases}$ = 5,474 $n_{controls}$ = 8,584) | Atherosclerotic cardiovascular diseases (CAD, MI, IS) | This SNP is associated with coronary artery disease. | [3] |
| **rs114694170 (T>C)** | Chr5: 88884379 (*MEF2C*) | Myogenesis transcription enhancer involved in cardiac morphogenesis, myogenesis and | GWAS Meta-analysis | 10 cohorts of European ancestry (n = 16,112) | Effect on VEGF levels | Lower serum VEGF-A levels in 9 out of 10 cohorts (T allele) | [9] |
| | | | Mendelian randomization study | 1000 Genomes data on adults of European ancestry | Ischemic Heart Disease | Potential contributor to VEGF phenotypic variance and IHD risk (C allele) | [10] |

| | | | | | | | |
|---|---|---|---|---|---|---|---|
| | | vascular development. | | ($n_{cases}$ = 60,801 $n_{controls}$ = 123,504) | | | |
| **rs6993770 (A>T)** | Chr8: 105569300 (*ZFPM2*) | Transcription regulator involved in heart morphogenesis and development of coronary vessels | GWAS Meta-analysis | 10 cohorts of European ancestry (n = 16,112) | Effect on VEGF levels | Increased serum VEGF-A levels in 9 out of 10 cohorts (A allele) | [9] |
| | | | Mendelian randomization study | 1000 Genomes data on adults of European ancestry ($n_{cases}$ = 60,801 $n_{controls}$ = 123,504) | Ischemic Heart Disease | Contributor to VEGF phenotypic variance by Increasing VEGF-A serum levels (A allele). The SNP is also associated with platelet count. | [10] |
| | | | Cross sectional population-based study | Lebanese unrelated participants (n = 460) | Influence on circulating lipid levels | This SNP is a contributor to VEGF-A circulating level heritability. It is also associated with total cholesterol, LDL, and hypercholesterolemia | [11] |
| | | | Case-control study | Iranian participants ($n_{cases}$ = 235, $n_{controls}$ = 101) | Metabolic syndrome | Association with fasting blood glucose, triglyceride levels and systolic blood pressure (T allele)<br>Increased risk for Metabolic Syndrome (AT genotype) | [12] |
| | | | GWAS community-based study | 3 healthy cohorts from USA (n = 3,527), France (n = 859), and Sweden (n = 868) | Effect on VEGF levels | Increased serum levels in 3 study groups (T allele) | [13] |
| | | | Population study | 2 groups of unrelated healthy European ancestry ($n_1$ = 1,006 $n_2$ = 1,145) | Influence on lipid metabolism | Increased HDL levels (T allele) | [14] |
| | | | Case-control study | Iranian participants ($n_{cases}$ = 248, $n_{controls}$ = 100) | Association between dietary intake and metabolic Syndrome | Association of metabolic syndrome with low dietary iron intake (TT genotype) | [15] |
| | | | Population study | Healthy French individuals (n = 403) | Association between VEGF, adhesion, and inflammation molecules. | An epistatic interaction (TA genotype and T allele) with two other SNPs is associated with Increased levels of ICAM-1, E-selectin, and IL-6 levels. | [16] |

| | | | | | | | |
|---|---|---|---|---|---|---|---|
| **rs2375981 (C>G)** | Chr9: 2692583 (*KCNV2*) | Codes for a potassium channel, regulates smooth muscle contraction and heart rate | GWAS Meta-analysis | 10 cohorts of European ancestry (n = 16,112) | Effect on VEGF levels | Increased serum VEGF-A levels in 10 cohorts (C allele) | [9] |
| | | | Population based phenome wide association study | Finnish participants (n = 6,890) | Inflammatory biomarker driver trait search | This SNP has a potential effect on VEGF-A protein production. It has been associated with IFN-γ, IL-10, IL-12p70 and VEGF-A molecules. | [17] |
| | | | Mendelian randomization study | 1000 Genomes data on adults of European ancestry ($n_{cases}$ = 60,801 $n_{controls}$ = 123,504) | Ischemic Heart Disease | Potential contributor to VEGF phenotypic variance and IHD (C allele) | [10] |
| | | | GWAS community-based study | 3 healthy cohorts from USA (n = 3,527), France (n = 859), and Sweden (n = 868) | Effect on VEGF levels | Lower serum VEGF-A levels in 3 study groups (G allele) | [13] |
| **rs7043199 (A>T)** | Chr9: 2621145 (*VLDLR*) | Low density lipoprotein receptor involved in endocytosis | GWAS Meta-analysis | 10 cohorts of European ancestry (n = 16,112) | Effect on VEGF levels | Lower serum VEGF-A levels in 10 cohorts (A allele) | [9] |
| | | | Mendelian randomization study | 1000 Genomes data on adults of European ancestry ($n_{cases}$ = 60,801 $n_{controls}$ = 123,504) | Ischemic Heart Disease | Potential contributor to VEGF phenotypic variance and IHD (T allele) | [10] |
| **rs10738760 (A>G)** | Chr9: 2691186 (*VLDLR* and *KCNV2*) | *VLDLR*: Low density lipoprotein receptor involved in endocytosis *KCNV2*: Potassium channel subunit that is predominantly expressed in the heart and retina | Cross sectional population-based study | Lebanese unrelated participants (n = 460) | Influence on circulating lipid levels | This SNP is a contributor to VEGF-A circulating level heritability. It also has potential implications in metabolic syndrome | [11] |
| | | | GWAS community-based study | 3 healthy cohorts from USA (n = 3,527), France (n = 859), and Sweden (n = 868) | Effect on VEGF levels | Increased VEGF-A serum levels in 3 study groups (A allele) | [13] |
| | | | Healthy cohort study | Lebanese general population (m = 460) | Effect of VEGF-A SNPs on iron levels | Association with low iron levels and obesity (GG genotype) | [18] |

| | | | | | | | |
|---|---|---|---|---|---|---|---|
| | | | Population study | Healthy French individuals (n = 403) | Association between VEGF, adhesion, and inflammation molecules. | An epistatic interaction of the G allele with two SNPs is associated with Increased ICAM-1 and E-selectin levels.<br>An epistatic interaction of the A allele with two other SNPs is associated with Increased VEGF-A and decreased IL-6 levels | [16] |
| **rs10761741 (T>G)** | Chr10: 63306426 (*JMJD1C*) | Histone demethylase involved in transcription regulation, RNA processing and DNA repair | GWAS Meta-analysis | 10 cohorts of European ancestry (n = 16,112) | Effect on VEGF levels | Increased serum VEGF-A levels in 10 cohorts (T allele) | [9] |
| | | | Mendelian randomization study | 1000 Genomes data on adults of European ancestry ($n_{cases}$ = 60,801 $n_{controls}$ = 123,504) | Ischemic Heart Disease | Potential contributor to VEGF phenotypic variance and IHD (T allele) | [10] |
| | | | Healthy cohort study | Middle-age men of European ancestry (n = 1,300) | Association with haemostatic factors | Increased mean platelet volume, decreased platelet count, and decreased platelet reactivity (G allele) | [19] |
| **rs4782371 (G>T)** | Chr16: 88502423 (*ZFPM1*) | Transcription regulator involved in cardiac development and platelet production | GWAS Meta-analysis | 10 cohorts of European ancestry (n = 16,112) | Effect on VEGF levels | Lower serum VEGF-A levels in 10 cohorts (T allele) | [9] |
| | | | Mendelian randomization study | 1000 Genomes data on adults of European ancestry ($n_{cases}$ = 60,801 $n_{controls}$ = 123,504) | Ischemic Heart Disease | This SNP is a potential contributor to VEGF phenotypic variance and IHD (G allele) | [10] |
| **rs2639990 (C>T)** | Chr18: 75203596 *(ZADH2)* | Protein that shows oxidoreductase and transferase activity predicted to be involved in negative regulation of fat cell differentiation. | GWAS Meta-analysis | 10 cohorts of European ancestry (n = 16,112) | Effect on VEGF levels | Increased serum VEGF-A levels in 10 cohorts (T allele) | [9] |
| | | | GWAS community-based study | 3 healthy cohorts from USA (n = 3,527), France (n = 859), and Sweden (n = 868) | Effect on VEGF levels | Increased VEGF-A serum levels in 3 study groups (T allele) | [13] |
| | | | Mendelian randomization study | 1000 Genomes data on adults of European ancestry ($n_{cases}$ = 60,801 $n_{controls}$ = 123,504) | Ischemic Heart Disease | This SNP is a potential contributor to VEGF phenotypic variance and IHD (T allele) | [10] |

*Location obtained from the Ensembl human database (GRCh38.p13).

$ Function of genes obtained from the Genecards database

CAD: Coronary Artery Disease. CHD : Coronary Heart Disease, GWAS: Genome Wide Association Study, HDL: High Density Lipoprotein, ICAM: Intercellular Adhesion Molecule, IHD: Ischemic Heart Disease, IL: Interleukin, IS: Ischemic Stroke, LDL: Low Density Lipoprotein, MI: Myocardial Infarction, sFlt: soluble fms-like tyrosine kinase-1 (sVEGFR1), sKDR: soluble Kinase insert Domain Receptor (sVEGFR2), SNP: Single Nucleotide Polymorphism, T2DM: Type 2 Diabetes Mellitus, USA: United States of America, VEGF: Vascular Endothelial Growth Factor

## References


1. Li, L., et al., *Association of Genetic Polymorphisms on Vascular Endothelial Growth Factor and its Receptor Genes with Susceptibility to Coronary Heart Disease.* Medical Science Monitor, 2016. **22**: p. 31-40.
2. Liu, D., et al., *Association of Genetic Polymorphisms on VEGFA and VEGFR2 With Risk of Coronary Heart Disease.* Medicine, 2016. **95**(19): p. e3413.
3. Wang, L., et al., *A meta-analysis of the relationship between VEGFR2 polymorphisms and atherosclerotic cardiovascular diseases.* Clin Cardiol, 2019. **42**(10): p. 860-865.
4. Zhang, L.J., et al., *Association of VEGFR-2 Gene Polymorphisms With Clopidogrel Resistance in Patients With Coronary Heart Disease.* Am J Ther, 2016. **23**(6): p. e1663-e1670.
5. Marks, E.C.A., et al., *Plasma levels of soluble VEGF receptor isoforms, circulating pterins and VEGF system SNPs as prognostic biomarkers in patients with acute coronary syndromes.* BMC Cardiovascular Disorders, 2018. **18**(1): p. 169.
6. Li, Z., et al., *Missense Variants in Hypoxia-Induced VEGFA/VEGFR2 Signaling Predict the Outcome of Large Artery Atherosclerotic Stroke.* Cellular and Molecular Neurobiology, 2021. **41**(6): p. 1217-1225.
7. Oh, S.-H., et al., *Association between kinase insert domain-containing receptor gene polymorphism and haplotypes and ischemic stroke.* Journal of the Neurological Sciences, 2011. **308**(1): p. 62-66.
8. Merlo, S., et al., *Vascular Endothelial Growth Factor Gene Polymorphism (rs2010963) and Its Receptor, Kinase Insert Domain-Containing Receptor Gene Polymorphism (rs2071559), and Markers of Carotid Atherosclerosis in Patients with Type 2 Diabetes Mellitus.* Journal of Diabetes Research, 2016.
9. Choi, S.H., et al., *Six Novel Loci Associated with Circulating VEGF Levels Identified by a Meta-analysis of Genome-Wide Association Studies.* PLOS Genetics, 2016. **12**(2): p. e1005874.
10. Au Yeung, S.L., H. Lam, and C.M. Schooling, *Vascular Endothelial Growth Factor and Ischemic Heart Disease Risk: A Mendelian Randomization Study.* Journal of the American Heart Association, 2017. **6**(8).
11. Salami, A. and S. El Shamieh, *Association between SNPs of Circulating Vascular Endothelial Growth Factor Levels, Hypercholesterolemia and Metabolic Syndrome.* Medicina, 2019. **55**(8).
12. Azimi-Nezhad, M., et al., *The Relationship Between Vascular Endothelial Growth Factor Cis- and Trans-Acting Genetic Variants and Metabolic Syndrome.* American Journal of the Medical Sciences, 2018. **355**(6): p. 559-565.
13. Debette, S., et al., *Identification of cis- and trans-acting genetic variants explaining up to half the variation in circulating vascular endothelial growth factor levels.* Circulation Research, 2011. **109**(5): p. 554-63.
14. Stathopoulou, M.G., et al., *A common variant highly associated with plasma VEGFA levels also contributes to the variation of both LDL-C and HDL-C.* Journal of Lipid Research, 2013. **54**(2): p. 535-41.
15. Hoseini, Z., et al., *VEGF gene polymorphism interactions with dietary trace elements intake in determining the risk of metabolic syndrome.* Journal of Cellular Biochemistry, 2019. **120**(2): p. 1398-1406.
16. Azimi-Nezhad, M., et al., *Associations of vascular endothelial growth factor (VEGF) with adhesion and inflammation molecules in a healthy population.* Cytokine, 2013. **61**(2): p. 602-7.
17. Ruotsalainen, S.E., et al., *An expanded analysis framework for multivariate GWAS connects inflammatory biomarkers to functional variants and disease.* European Journal of Human Genetics, 2021. **29**(2): p. 309-324.

18. Chedid, P., et al., *The association of vascular endothelial growth factor related SNPs and circulating iron levels might depend on body mass index.* Frontiers in Bioscience-Landmark, 2022. **27**(1).
19. Eicher, J.D., et al., *Replication and hematological characterization of human platelet reactivity genetic associations in men from the Caerphilly Prospective Study (CaPS).* J Thromb Thrombolysis, 2016. **41**(2): p. 343-50.
